# The *Chlamydia trachomatis* Protease CPAF Contains a Cryptic PDZ-Like Domain with Similarity to Human Cell Polarity and Tight Junction PDZ-Containing Proteins

**DOI:** 10.1371/journal.pone.0147233

**Published:** 2016-02-01

**Authors:** Kenneth R. Maksimchuk, Katherine A. Alser, Rui Mou, Raphael H. Valdivia, Dewey G. McCafferty

**Affiliations:** 1 Department of Biochemistry, Duke University Medical Center, Durham, North Carolina, United States of America; 2 Department of Chemistry, Duke University, Durham, North Carolina, United States of America; 3 Molecular Genetics and Microbiology, Duke University, Durham, North Carolina, United States of America; University of the Pacific, UNITED STATES

## Abstract

The need for more effective anti-chlamydial therapeutics has sparked research efforts geared toward further understanding chlamydial pathogenesis mechanisms. Recent studies have implicated the secreted chlamydial serine protease, chlamydial protease-like activity factor (CPAF) as potentially important for chlamydial pathogenesis. By mechanisms that remain to be elucidated, CPAF is directed to a discrete group of substrates, which are subsequently cleaved or degraded. While inspecting the previously solved CPAF crystal structure, we discovered that CPAF contains a cryptic N-terminal **P**SD95 **D**lg **Z**O-1 (PDZ) domain spanning residues 106–212 (CPAF^106-212^). This PDZ domain is unique in that it bears minimal sequence similarity to canonical PDZ-forming sequences and displays little sequence and structural similarity to known chlamydial PDZ domains. We show that the CPAF^106-212^ sequence is homologous to PDZ domains of human tight junction proteins.

## Introduction

*Chlamydia trachomatis* is one of the leading bacterial pathogens, infecting over 100 million people worldwide annually [1]. Four million people, in the US alone, are affected each year despite the aggressive implementation of antibiotic treatment regimens and public health awareness campaigns [2]. *C*. *trachomatis* is an obligate intracellular pathogen, with fifteen different serovars affecting various mucous membranes in the body. *C*. *trachomatis* invades epithelial tissues including those in the eyes and reproductive tract, where it affects the single outer layer of columnar cells. Currently, azithromycin and doxycycline are the antibiotics of choice to combat chlamydial infections. However, because infections are asymptomatic in nearly 70 percent of cases, patients frequently fail to seek treatment [3, 4]. As a result, secondary maladies such as scarring, ectopic pregnancy, infertility, and blindness are frequently associated with chronic and recurring infection [5]. Based on its high transmission rate and the frequency of treatment failure, *C*. *trachomatis* stands to pose a significant public health threat in the near future, similar to that currently posed the recent drug-resistant *Neisseria gonorrhoeae*.

*C*. *trachomatis* has a biphasic developmental cycle, in which infectious elementary bodies (EB) invade host cells. From there, the resulting endosomal compartment evades lysosomal degradation pathways and forms an intracellular parasitophorous vacuole termed an inclusion. From within this inclusion, *C*. *trachomatis* is effectively cloaked and protected from host immunological defenses. Chlamydial EB differentiate into metabolically active reticulate bodies (RB) that undergo replication and initiate protein synthesis [6]. During this stage of infection, *C*. *trachomatis* secretes effectors into the host cytoplasm that perform numerous functions to maintain the infected cell in a state where the immune defenses are dampened, cell viability is enhanced, and pro-apoptotic signaling is inhibited [7–10]. The RB transition back into EB during late stages of infection and ultimately, the pathogen induces cell lysis or inclusion extrusion to initiate new infection cycles in neighboring cells.

Chlamydial effector proteins are translocated into the host cell cytoplasm and dynamically remodel the inclusion, interfere with host cell apoptosis, dampen immune responses, and inflammation, as well as abrogate or co-opt signal transduction pathways. Of the known chlamydial effectors, the serine protease chlamydial protease-like activity factor (CPAF) has been identified as a factor involved in the intracellular biology of *Chlamydia* that cleaves host proteins including the cytoskeleton intermediate filament vimentin, the nuclear envelope lamin-associated protein (LAP1), and also disrupts cell division [11–15]. However, the molecular mechanisms by which CPAF disrupts these pathways remain elusive.

Assembly of many functional signal transduction complexes are facilitated by protein-protein interaction motifs such as **P**SD95 **D**lg **Z**O-1 (PDZ), **S**rc **H**omology **2** (SH2), **S**rc **H**omology 3 (SH3), and WW domains [16]. In particular, PDZ domains mediate a significant proportion of these interactions. The PDZ domain frequently serves as a receptor for C-terminal tetrapeptide sequences in tail-specific proteases like HtrA, DegP, photosystem II D1 protein peptidase and other hydrolases [17–20]. PDZ domains in signaling and scaffolding proteins mediate protein-protein interactions through capture of C-terminal tetrapeptide sequences, internal peptide sequences, or through PDZ-to-PDZ domain interactions [21, 22]. PDZ domain interactions are critical components of cellular tight junctions, signal transduction pathways, inflammation responses and assembly of immune complexes [23–25]. Additionally, viral pathogens are known to mimic host PDZ ligands and act as decoys to disrupt protein-protein interactions [22, 26].

While inspecting the CPAF crystal structure, we discovered that CPAF contains a cryptic N-terminal PDZ domain. This PDZ domain is unique in that it bears almost no sequence similarity to canonical PDZ-forming sequences and displays minimal sequence and structural similarity to known chlamydial PDZ domains. We found that the CPAF^106-212^ sequence is most similar to PDZ domains of human proteins involved in cell polarity and epithelial tight junction formation.

## Materials and Methods

All protein structures were retrieved from the RCSB Protein Data Bank repository [27]. Upon inspection of the mature CPAF crystal structure (PDB ID: 3DOR) [28], we hypothesized that the enzyme contains what appears to be a PDZ-like fold spanning residues 106–212 (PDB 3DOR residues 114–220) in the N-terminal domain (CPAF^106-212^). The coordinates corresponding to residues CPAF^106-212^ were extracted from the mature CPAF structure and analyzed by the methods outlined below.

### Structural Similarity of CPAF^106-212^ to Canonical PDZ Domains

The CPAF^106-212^ structure was compared to those of canonical PDZ domains known to bind to class I, II, and III ligands, as well as to those of unclassified PDZ domains (PDB ID): **(Class I)** PSD95-PDZ3^306-402^ (1TQ3, 306–402) [29], NHERF2-PDZ1^9-91^ (2OCS, 8–90) [30], syntenin-PDZ1^112-193^ and syntenin-PDZ2^195-273^ (1N99, 112–193 and 195–273) [31], and erbin^1318-1412^ (1MFG, 1277–1371) [32]; **(Class II)** CASK^487-572^ (1KWA, 487–552) [33], erythrocyte p55^69-153^ (2EV8, 69–153) [34], HtrA1^380-480^ (2JOA, 380–480) [35], and HtrA2^362-454^ (1LCY, 229–321) [36]; **(Class III)** nNOS^14-100^ (1QAU, 14–100) [37], ABPA1-PDZ1^653-741^ (1U37, 17–105) [38], and afadin^1001-1096^ (1XZ9, 1001–1096) [39]; **(Unclassified)** PS2-D1P^158-249^ (1FC6, 158–249) [19], DegP-PDZ1^287-379^ (3MH4, 261–353) [40], MPP7^136-219^ (3O46, 136–219) [41], Shroom4^6-92^ (2EDP, 8–94) [42] and HtrA3^354-453^ (2P3W, 354–453) [35].

Each set of PDZ domain coordinates was extracted from the full PDB coordinates and aligned to CPAF^106-212^ using the STructural Alignment of Multiple Proteins (STAMP) feature of the Visual Molecular Dynamics (VMD) software suite [43], as well as the pairwise DaliLite server [44]. Root mean square distance (RMSD) and percent identity (PID) values were obtained for each superposition for each of the methods above. The results were then analyzed together to determine structural similarity of CPAF^106-212^ to the representative canonical PDZ domains.

### Identification of Structural CPAF^106-212^ Homologs

The PDB coordinates for CPAF^106-212^ were submitted to the Dali Protein Structure Database server [44] in order to identify proteins containing similar structural elements. The results of the Dali search yielded 749 protein chains with Z-scores greater than 2.0, indicating significant structural homology. Following removal of duplicate PDB identifiers, these hits corresponded to 437 unique protein structures that exhibited an average Dali Z-score of 4.9 ± 1.9. Values for RMSD, PID and alignment length were recorded. A total of 147 structures with RMSD values ≤ 2.5 Å were carried forward for further analysis. These hits were cross-referenced with the UniProtKB database [45] to retrieve 85 corresponding unique protein identifiers.

The UniProtKB accession numbers for the resulting proteins were cross-referenced with the NCBI Conserved Domain Database (CDD) [46] to map all known protein domains and identify proteins that contained at least one annotated PDZ domain. The NCBI CDD search was carried out using the CDD search option with 0.01 expect value and composition corrected scoring. Retired sequences were also included in the search. A total of 65 unique protein accession numbers were indicated to contain at least one annotated PDZ domain, several of which contained multiple PDZ domains. Of the 65 proteins, we noted 42 (65%) from *Homo sapiens*, 13 (20%) from mouse and rat, 3 (5%) from *Escherichia coli*, and 2 (3%) from *Drosophila melanogaster*.

### Bioinformatic Data Mining to Investigate CPAF^106-212^ Sequence Homology

The 65 proteins mapped to 170 PDZ domain sequences, which were retrieved from the UniProtKB database and limited to the residue ranges provided by the CDD search. The PDZ domain sequences were aligned against CPAF^106-212^ using the NCBI Protein BLAST (blastp) server [47, 48] to identify PDZ domains with high sequence homology. A total of 29 sequences scored E-values less than 0.001 and bit scores above 20.0. Sequences that corresponded to multiple hits within the same protein family were submitted for multiple sequence alignment with Clustal Omega [48, 49]. The resulting alignments were annotated using ESPript 3.0 [50] for figure generation.

### Assessment of PDZ Occurrence in the *C*. *trachomatis* Genome

We searched across all chlamydial strains and species for proteins containing annotated PDZ domains using the Pfam database [51]. The results were filtered by species and PDZ family (pf000595, pf13160 and pf14685). Species-specific PDZ occurrence was recorded as the number of PDZ domains in one chlamydial species compared to the total number of PDZ domains observed among all species of *Chlamydia*. The chlamydial PDZ domains were cross-referenced against the UniProtKB database and the proposed function(s) for each protein was obtained from the database.

## Results

**Fig 1:**
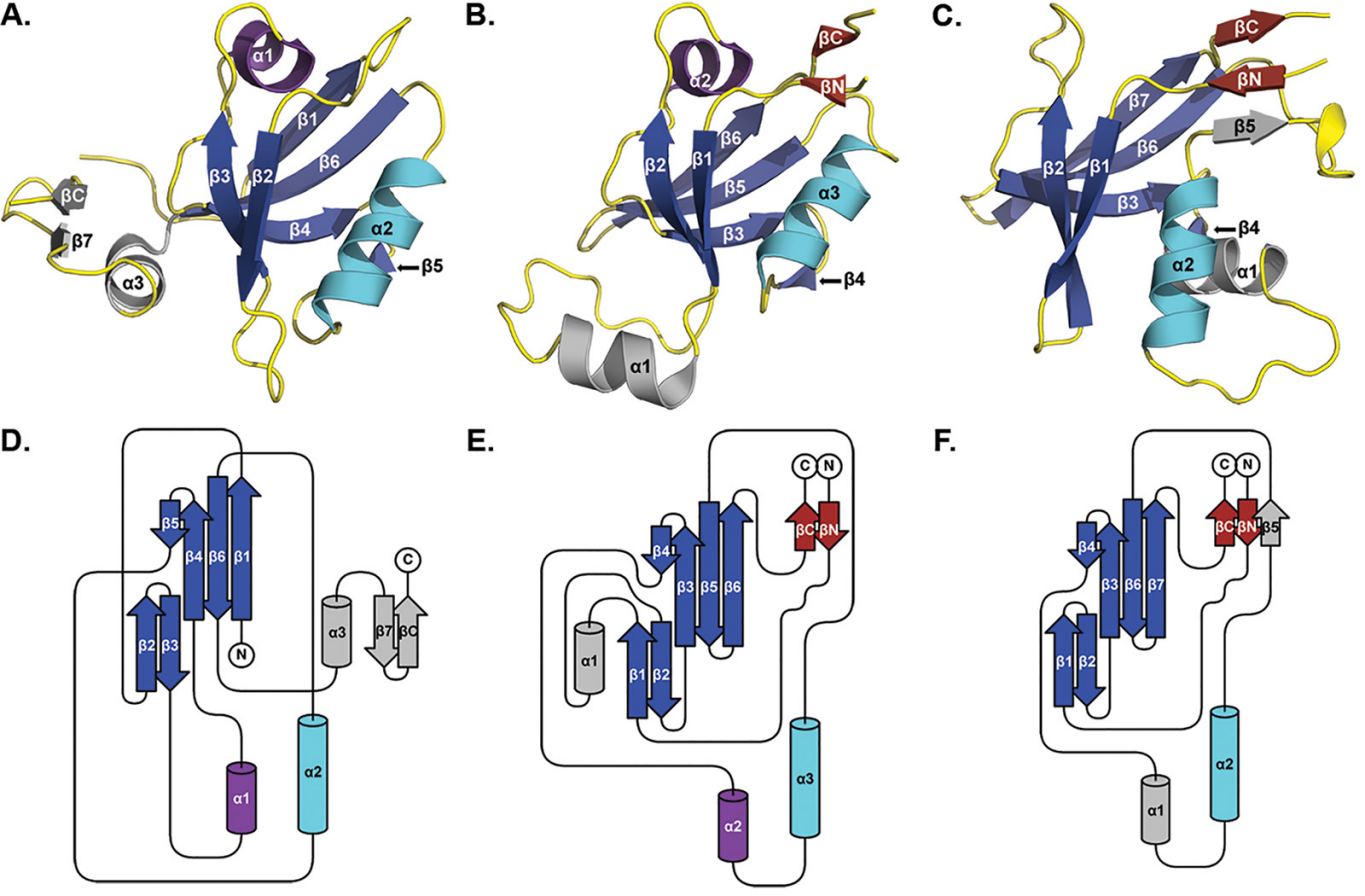
CPAF^106-212^ Shares a Permutation of the Common PDZ Fold from Canonical PDZ Domains from PSD95-PDZ3 and HtrA2. PDZ domains from PSD95-PDZ3 (Class I, PDB ID: 1TQ3) **(A)**, HtrA2 (Class II, PDB ID: 1LCY) **(B)** and CPAF (PDB ID: 3DOR) **(C)** are depicted with common PDZ elements colored. The core five-stranded beta sheet is shown in dark blue, and the ligand-binding domain alpha helix in cyan. The ligand-binding pocket is formed by β2 and α2 in PSD95-PDZ3, and by β1 and α3 in HtrA2. β1 and α2 in CPAF represents the predicted ligand-binding domain. A two-stranded beta sheet is conserved between HtrA2 and CPAF, shown in red. Elements not shared between the three domains are shown in gray. Domain maps for PSD95-PDZ3 **(D)**, HtrA2 **(E)**, and CPAF **(F)** show a common domain arrangement between the three PDZ domains that only differ in the connectivity of disordered loops.

Upon inspection of the mature CPAF crystal structure, we observed that the enzyme contains a PDZ-like fold spanning residues 106–212 in the N-terminal domain (CPAF^106-212^). Canonical PDZ domains share a common fold consisting of five antiparallel beta strands and two alpha helices, in which the ligand-binding pocket is formed by the β2 strand and α2 helix, as depicted by the class I PDZ domain from PSD95 (PSD95-PDZ3) (Fig 1A). Variations on this architecture are observed in which additional helices or strands decorate the PDZ domain, as demonstrated by both the class II domain from HtrA2 as well as the CPAF^106-212^ domain (Fig 1B and 1C). Additionally, the components of the PDZ domain can be connected by flexible loops in various configurations, a phenomenon called circular permutation [52]. This phenomenon of diverse domain connectivity and conservation of the overall PDZ fold is demonstrated by the PDZ domain topology maps of PSD95-PDZ3, HtrA2 and CPAF (Fig 1D–1F).

### Structural Similarity of CPAF^106-212^ to Canonical PDZ Domains

The residues lining the ligand-binding pocket of PDZ domains contribute significantly to peptide ligand binding and specificity. Thus, sequence similarity along the PDZ binding site hotspots is frequently a strong predictor of ligand specificity [21, 53]. Canonical PDZ domains are generally grouped into three major classes based on which types of ligands they bind: class I ligands (X-[S/T]-X-Φ), class II ligands (X-Φ-X-Φ), or class III ligands (X-D/E/K/R-X-Φ), where X is any residue and Φ represents hydrophobic residues (V, I, L, M, F, W and Y) [22].

To examine if the putative CPAF PDZ domain structure was consistent with a canonical PDZ-like fold, we used VMD-STAMP [43] and the pairwise DaliLite server [44] to compare CPAF amino acid residues 106 to 212 (CPAF^106-212^) to representative structures of all three canonical PDZ domain classes, as well as to unclassified domains (Table 1). Values for root mean square deviation (RMSD) and percent identity (PID) were recorded for all comparisons, and the results were analyzed together. RMSD and PID values obtained using VMD-STAMP result from alignment of all residues in the compared structures, and thus provide a more conservative basis for analysis of similarities. The RMSD and PID values obtained from the pairwise DaliLite server result from alignment of the shared common structure, thus accounting for differences resulting from loop mobility, helix torsions, and other minor structural variations.

**Table 1:** Comparison of CPAF^106-212^ to Canonical and Unclassified PDZ Domains.

Class	PDZ Domain	Residues	STAMP-VMD		DALI	
			RMSD^a^ Å	PID^b^ %	RMSD^a^ Å	PID^b^ %
--	CPAF	106–212	--	--	--	--
**Class I**	PSD95-PDZ3	306–402	3.1	9	3.1	18
**(X-[S/T]-X-Φ)**	NHERF2-PDZ1	9–91	3.5	9	2.6	18
	Syntenin-PDZ1^c^	112–193	2.6	8	2.2	15
	Syntenin-PDZ2^d^	195–273	3.2	7	2.6	14
	Erbin^**b**^	1318–1412	2.8	7	2.1	17
	**Averages:**		**3.0**	**8**	**2.5**	**16**
**Class II**	CASK	487–572	2.9	12	2.7	23
**(X-Φ-X-Φ)**	Erythrocyte p55	69–153	3.0	12	2.3	23
	HtrA1	380–480	3.2	7	2.8	11
	HtrA2	362–454	2.5	10	2.4	18
	**Averages:**		**2.9**	**10**	**2.6**	**19**
**Class III**	nNOS	14–100	2.8	11	2.5	20
**(X-D/E/K/R-X-Φ)**	ABPA1-PDZ1	653–741	2.9	9	2.3	17
	Afadin (AF6)	1001–1096	3.2	9	2.9	21
	**Averages:**		**3.0**	**9**	**2.6**	**19**
**Unclassified**	PS2-D1P	158–249	2.6	10	2.5	15
	DegP-PDZ1	287–379	3.0	11	2.6	17
	MPP7	136–219	2.6	18	2.3	33
	Shroom4	6–92	2.9	12	2.4	24
	HtrA3	354–453	2.8	10	2.4	16
	**Averages:**		**2.8**	**12**	**2.4**	**21**

Structure-based alignment was performed using the STAMP module of the VMD software suite, and the pairwise DaliLite server. RMSD, and PID statistics were compared between CPAF^106-212^ and several PDZ domains of all three major classes, in addition to unclassified domains. The binding specificity of each PDZ domain class is indicated in parenthesis where X is any residue and **Φ** represents hydrophobic residues (V, I, L, M, F, W and Y).

^a^RMSD = Root Means Squared Deviation

^b^PID = Percent Identity

^c^In addition to class I ligands, these domains also bind class II ligands

^d^In addition to class I ligands, these domains also bind class III ligands

Both sets of data show low average RMSD values and high average PID values between CPAF^106-212^ and known PDZ domains. These data suggest that CPAF^106-212^ bears structural similarity known PDZ domains. Within this group of PDZ domains, CPAF^106-212^ had the lowest RMSD coupled with the highest PID when compared to the PDZ domain of human protein MPP7.

### Identification of CPAF^106-212^ Structural Homologs

**Fig 2:**
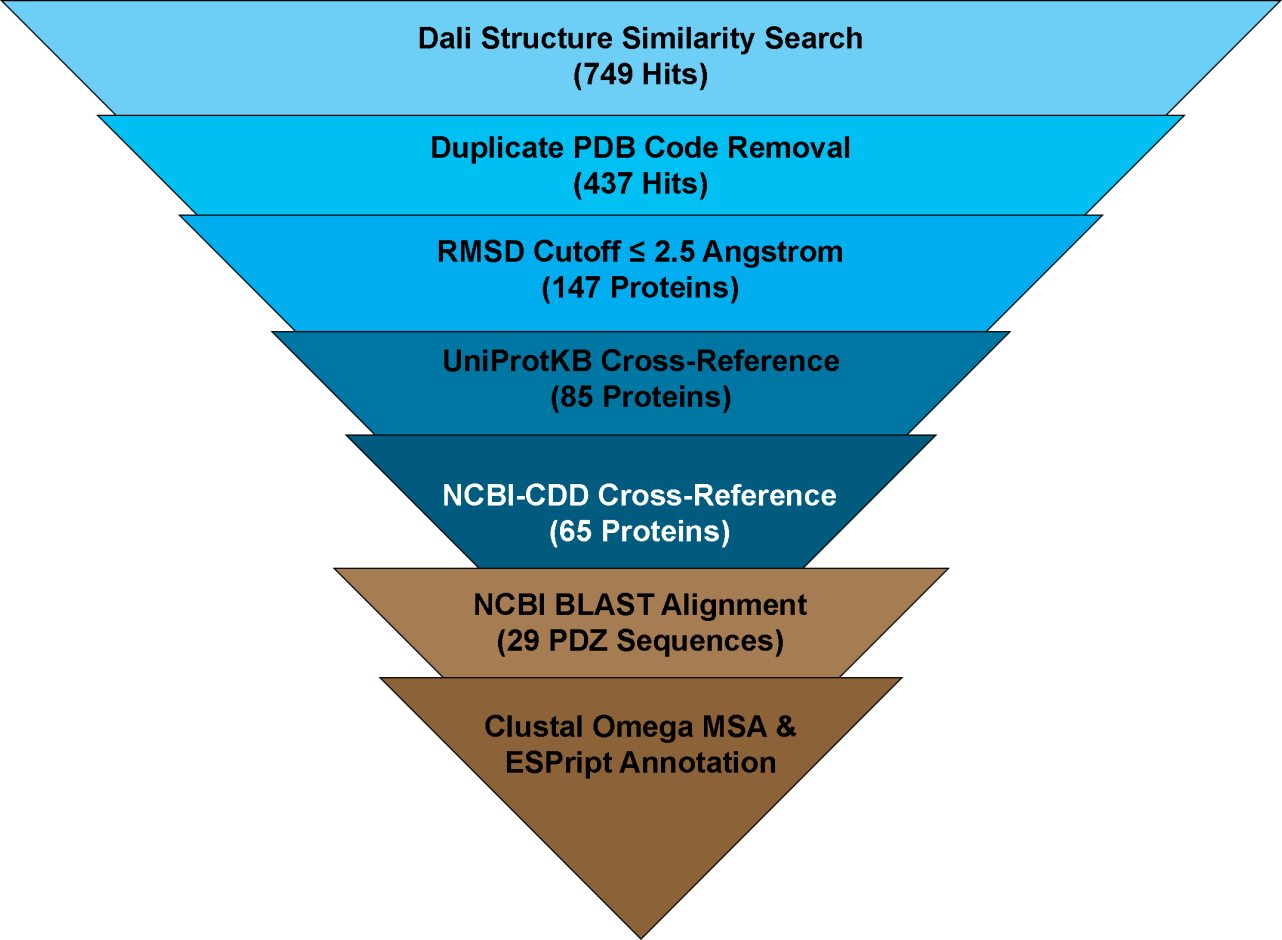
Bioinformatic Analysis of CPAF^106-212^. A multifaceted approach to identifying PDZ domains with high sequence and structural similarity to CPAF^106-212^ was adopted. From the mature CPAF enzyme crystal structure, residues 106–212 were isolated and submitted to bioinformatics webservers and the results filtered to retain only the PDZ domains with the highest similarity. Ultimately, 65 proteins with significant structural homology were identified and analyzed for sequence similarity.

In order to identify additional PDZ domains with structural homology to CPAF^106-212^, we submitted the PDB coordinates for CPAF^106-212^ to the Dali protein structure database server [44], and processed the data as follows (Fig 2). The Dali webserver is designed to compare a single protein structure against all solved protein structures in the RCSB database and evaluates the individual comparisons for strong structural homology. As a result, the Dali webserver provides a comprehensive structural comparison of the putative CPAF PDZ domain against other PDZ-containing proteins across all species. We obtained a dataset of 437 unique PDB files that exhibited an average Z-score of 4.9 ± 1.9, where a Z-score of 2.0 represents significant structural homology. We reviewed the RMSD and PID values for the hits compared against CPAF^106-212^ and found an average RMSD and PID of 2.7 Å and 17 percent respectively.

The unique Dali results were filtered using an RMSD cutoff of 2.5 Å, yielding 147 PDB codes. We cross-referenced the unique PDB codes with the UniProtKB database [45] to retrieve protein accession numbers for the structures similar to CPAF^106-212^. There were a total of 85 unique identifiers retrieved, 52 of which were from *Homo sapiens* and 17 of which were of murine origin. Interestingly, this combined set of human and murine proteins comprise over 80 percent of the total hits and are also the two host targets of *C*. *trachomatis* and *C*. *muridarum*. Such a high incidence of human and murine proteins could be expected if CPAF utilizes a PDZ-dependent pathogenesis mechanism in these hosts. In fact, when sequences of CPAF homologues from the various species of *Chlamydia* are compared, the region corresponding to the putative PDZ domain is very highly conserved (S1 Fig). Additionally, several other proteins were of viral origin; including influenza, rabies, and human papilloma virus. These viral pathogens have been previously shown to use PDZ mimicry to manipulate cellular tight junctions and adherens junctions and to facilitate infection [21, 26].

The dataset of 85 UniProtKB identifiers was cross-referenced with the NCBI Conserved Domain Database (CDD) [46] to identify all of the annotated protein domains represented by our dataset. Within the list of candidate proteins, PDZ domains alone represented 41 percent of all specific and superfamily domains detected; the next highest annotated domain registered 3.6 percent. From these results we identified 65 unique UniProtKB accession codes that contained one or more PDZ domains, corresponding to a hit rate of 76 percent. Of the 65 candidate proteins, there were many that contained multiple PDZ domains. Following removal of superfamily and nonspecific hits to limit false positives, the resulting 170 PDZ domain sequences were analyzed further.

### Sequence Comparison to Identify CPAF^106-212^ Homologs

**Fig 3:**
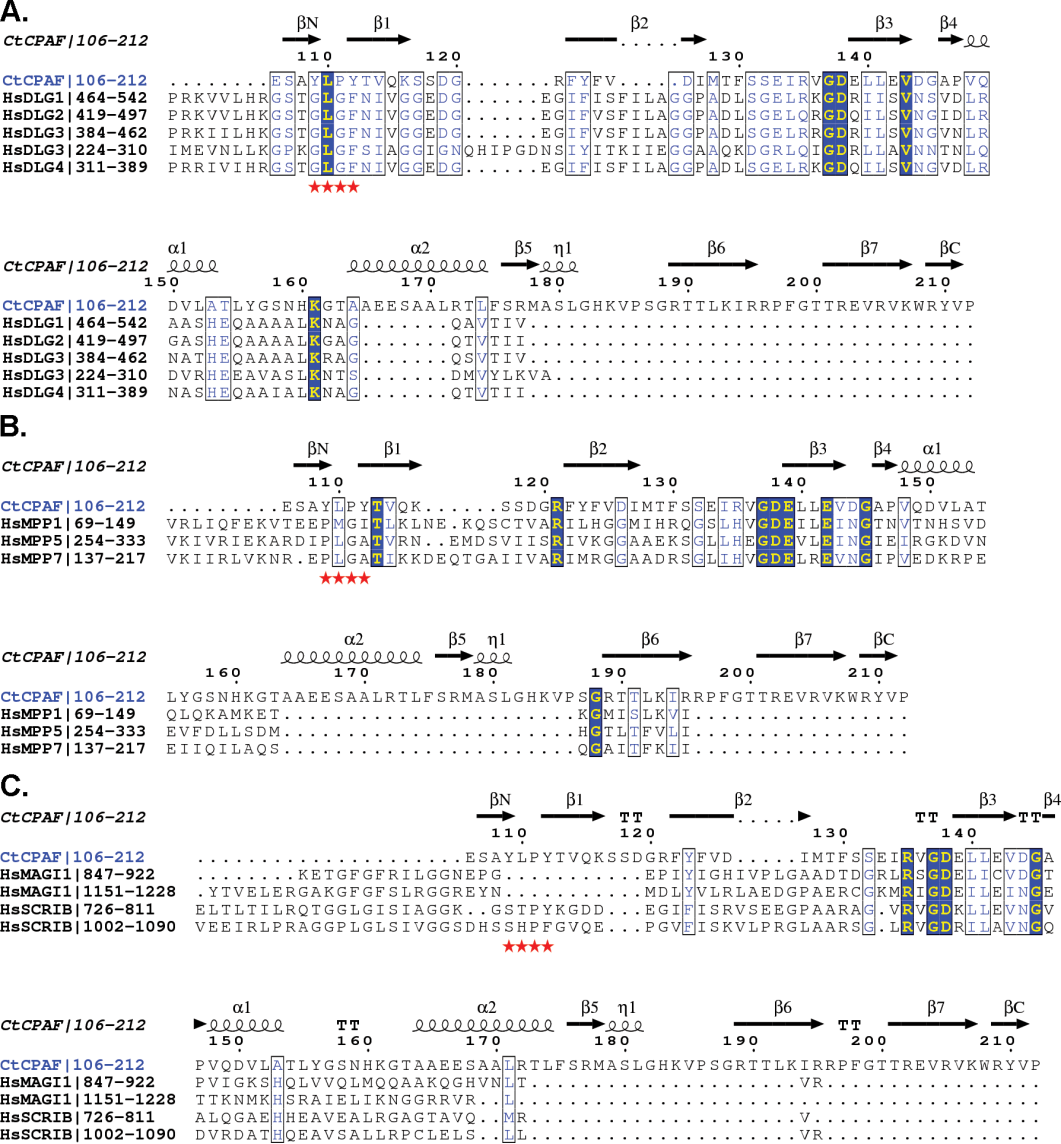
Sequence Alignment of CPAF^106-212^ Against Human PDZ Domains Involved in Epithelial Tight Junction Maintenance. Dlg **(A)** MPPx **(B)** MAGI1 and SCRIB **(C)** PDZ domains were aligned against CPAF^106-212^ using NCBI BLAST and Clustal Omega. The resulting annotations were performed with ESPript 3.0. Similar residues are depicted in blue/white while identically conserved residues are in yellow/blue. The canonical GLGF-loop for PDZ domains is annotated by red stars.

Having identified a set of proteins containing PDZ domains with high structural homology to CPAF^106-212^, we aimed to address to what extent these domains retain sequence homology with the CPAF PDZ domain. The 170 PDZ sequences that were previously identified were retrieved from the UniProtKB database using residue ranges supplied by the NCBI CDD. These sequences were submitted for alignment against CPAF^106-212^ using the NCBI Protein BLAST (blastp) server [47, 48]. We analyzed the bit scores and E-values of the resulting alignments and identified a cluster of hits with E-values less than 0.001 and bit scores above 20.0 (S2 Fig). The number of alignments necessary to achieve a bit score of 20 by random chance is approximately 5 fold greater than our search space for the 170 PDZ sequences, suggesting that these sequences display bona fide homology. Furthermore, the E-value filter ensures that there is minimal chance that a given result will have similar bit scores by random chance. Therefore, sequences that scored E-values less than 0.001 and bit scores above 20.0 were interpreted as having significant sequence homology to CPAF^106-212^. These sequences were submitted for multiple sequences alignment using the Clustal Omega webserver [49], and the resulting alignments were annotated using ESPript 3.0 [50] (Fig 3).

**Fig 4:**
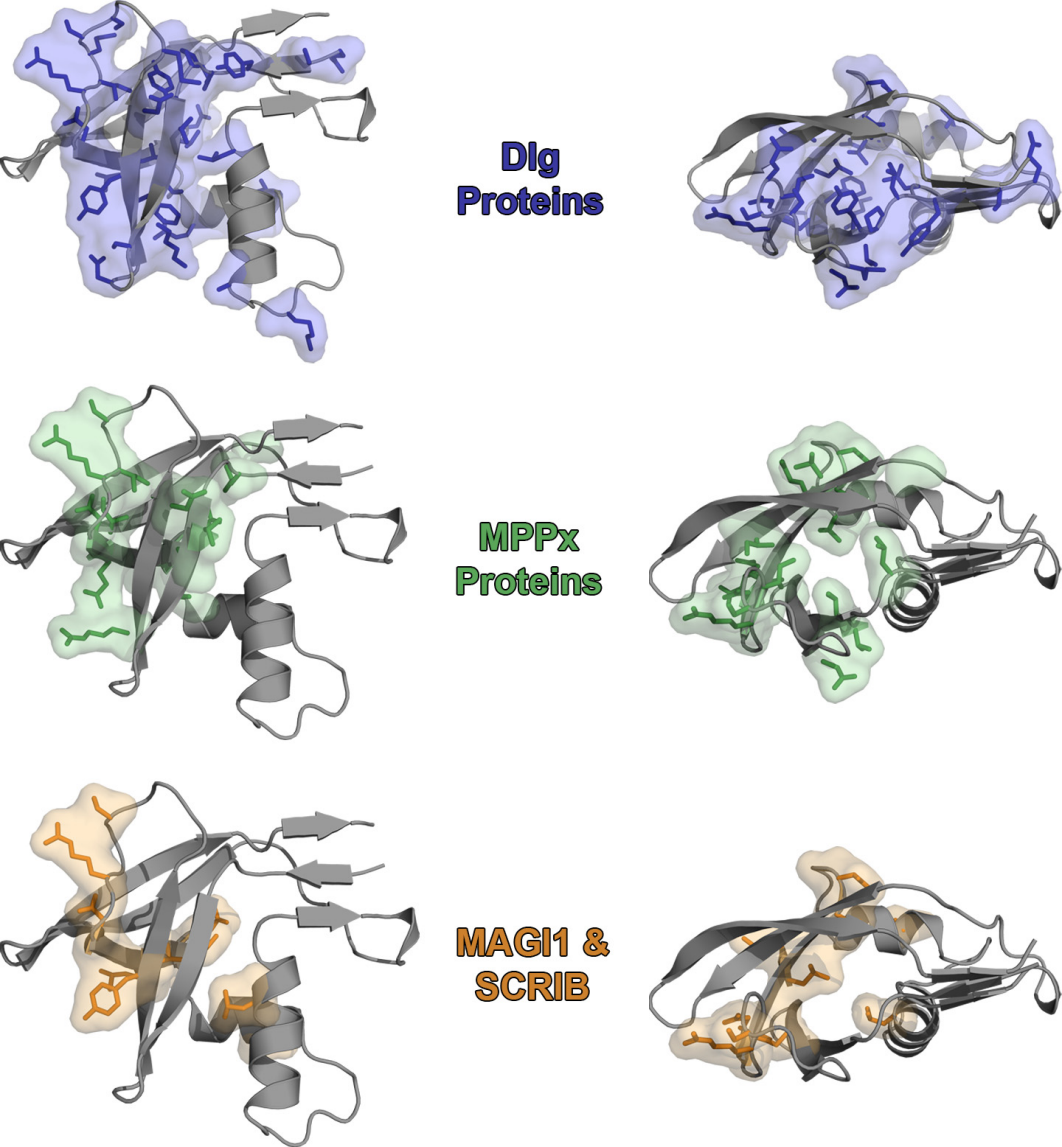
The Conserved Residues of the CPAF and Human Tight Junction PDZ Domains Map to the Core of the CPAF PDZ Domain. The residues highlighted as similar or conserved in Fig 3 were mapped onto CPAF^106-212^ (PDB ID: 3DOR) for each protein family. In each case, these residues for the Dlg1 proteins (top), MPPx proteins (middle), and the MAGI1 and SCRIB proteins (bottom) reside toward the core of the CPAF PDZ domain.

Epithelial tight junction and cell polarity proteins Dlg1-4, MPP1, 5, 7, MAGI1, and SCRIB have strong sequence similarity to CPAF^106-212^ within the PDZ domain recognition pocket. The conserved residues lie toward the core of the CPAF PDZ domain and binding pocket and may suggest that the residues are involved in establishing the PDZ fold to mimic host domains or in recognizing PDZ ligands (Fig 4). We speculate that, upon release into the cytosol, CPAF may be able to compete for PDZ ligands to disrupt host cell signaling. Alternatively CPAF^106-212^ may serve to deliver the protease to the same location as the Dlg, MPPx, MAGI1, or SCRIB proteins and their cognate ligands.

### Assessment of PDZ Occurrence in the *C*. *trachomatis* Genome

Having identified a cryptic PDZ domain in CPAF, we used the Pfam database to address whether PDZ domains are commonly annotated in chlamydial species and to which proteins they are coupled. We searched the Pfam database for known chlamydial PDZ domain types (pf000595, pf13160 and pf14685) and identified 86 unique PDZ-containing proteins from all species of *Chlamydia*. Of these proteins, 40 (46.5%) originate from *C*. *trachomatis*, making it the chlamydial species with the highest PDZ domain occurrence. Interestingly, all 40 of the PDZ-containing proteins identified in *C*. *trachomatis* are classified as hydrolases, in particular tail-specific and HtrA class peptidases.

We have discovered through bioinformatics analysis that CPAF contains what represents a cryptic PDZ domain within the N-terminal subunit of the enzyme, spanning residues 106–212. When compared to canonical exemplary domains from each major PDZ class, CPAF^106-212^ was shown to exhibit significant structural homology with unclassified PDZ domains, particularly that of human MPP7. The CPAF PDZ domain also shares structural and sequence homology with human PDZ domain-containing proteins involved in epithelial tight junction maintenance, including those from the MAGUK (Dlg, MPPx, and MAGI1) and LAP (SCRIB) families.

## Discussion

CPAF is a secreted S41 protease in the chlamydial arsenal that recognizes a broad range of substrates within the active site. S41 proteases that exhibit broad active site substrate specificity will frequently utilize secondary specificity elements to restrict enzyme activation or catalysis to a defined subset of targets. This phenomenon is demonstrated by proteins in the tail-specific, DegP and HtrA protease families. In many of the S41 proteases, including DegP, HtrA2, HtrA3, and tail-specific proteases, secondary regulation of enzyme activity is achieved through communication between the catalytic serine protease domain and a nearby PDZ domain [35, 54, 55]. Because CPAF contains similarly arranged catalytic and PDZ domains, we hypothesize that the CPAF PDZ domain may be involved in enzyme activation or substrate recognition.

**Fig 5:**
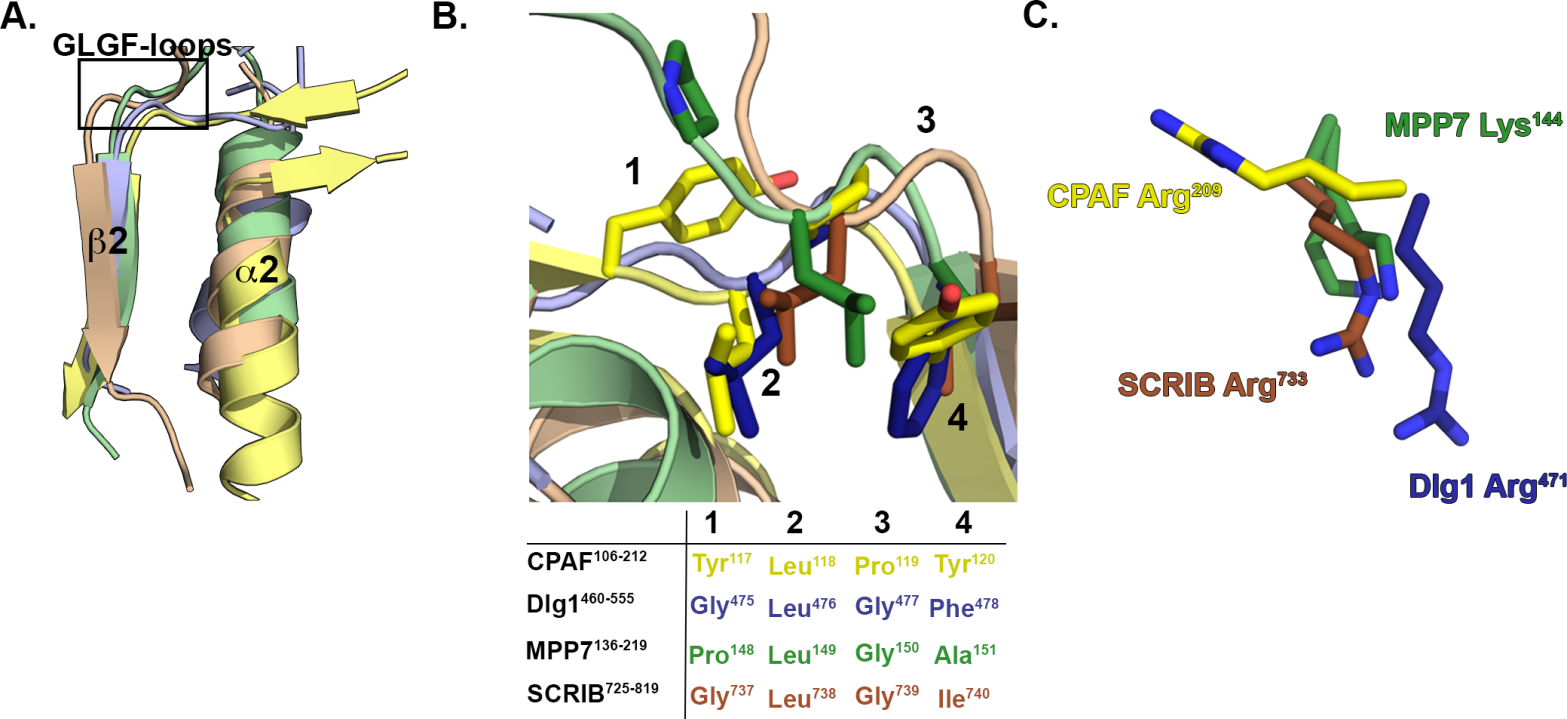
The CPAF YLPY-loop is Analogous to the GLGF-loop in Canonical PDZ Domains. **(A)** The PDZ binding site secondary structures, traditionally labeled β2 and α2, are shown for CPAF (PDB ID: 3DOR, yellow) overlaid with human PDZ domains from Dlg1 (PDB ID: 1PDR, blue) [56], MPP7 (PDB ID: 3O46, green), and SCRIB (PDB ID: 2W4F, brown) [57]. The location of the GLGF-loop is boxed. **(B)** The zoomed view of the GLGF-loop residues for CPAF, Dlg1, MPP7, and SCRIB PDZ domains, with each position annotated by a number for clarity. The GLGF-loop sequences for each domain are shown below the picture. **(C)** The isolated, conserved basic residues for Dlg1, MPP7, and SCRIB are overlaid with non-conserved CPAF Arg^209^ and shown to occupy the same space.

Canonical PDZ domains that recognize C-terminal tetrapeptides frequently contain a Gly-Leu-Gly-Phe (GLGF) motif in the binding site loop for ligand recognition (Fig 5A). The CPAF PDZ domain shares some sequence similarity with PDZ domains of human proteins in the MAGUK (Dlg, MPPx, and MAGI1) and LAP (SCRIB) families. Sequence alignment of these PDZ domains revealed that the analogous sequence in CPAF is unique and atypical of canonical PDZ motifs. In particular, the corresponding GLGF-loop in CPAF^106-212^ is composed of a Tyr^109^-Leu^110^-Pro^111^-Tyr^112^ (YTPY). This YTPY sequence maintains the typical hydrophobic nature of the GLGF-loop despite the fact that the sequence is divergent from canonical PDZ domains (Fig 5B).

Additionally, many canonical PDZ domains contain a conserved basic residue that is involved in binding C-terminal carboxyl groups of target peptides [56, 58, 59]. Though not evident in the sequence alignments, structural comparison of CPAF^106-212^ to the PDZ domains in human Dlg1, MPP7, and SCRIB revealed that Arg^209^ occupies an analogous region for the conserved basic residues from known PDZ domains (Fig 5C). The fact that Arg^209^ was not shown to align with the conserved basic residues of other PDZ domains suggests that CPAF may achieve parallel functionality within the PDZ domain through a circular permutation of its sequence. Mutational analysis of the YLPY-loop and Arg^209^ is necessary to confirm their roles in CPAF PDZ function and ligand recognition.

**Fig 6:**
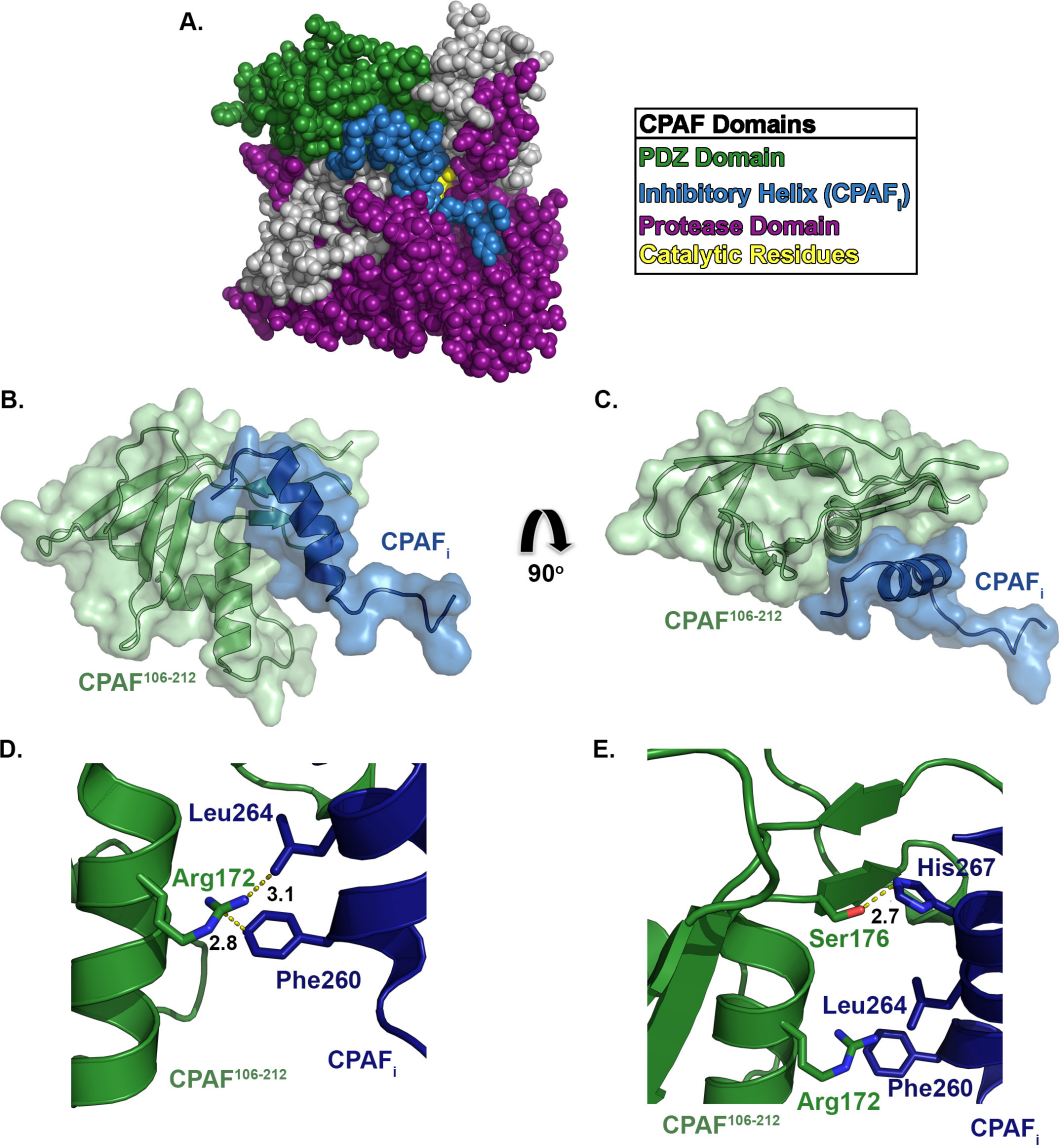
The CPAF Inhibitory Helix Makes Substantial Contact with the PDZ Domain in the Inactive Zymogen. **(A)** The domain organization of the CPAF zymogen (PDB ID: 3DPN) [28] shows that the inhibitory helix (CPAF_i_) resides in the active site groove and contacts both the protease and PDZ domains. **(B)** CPAF^106-212^ (green) is shown making multiple contacts with CPAF_i_ (blue). **(C)** Rotation of the zymogen structure allows for visualization of the contact between the PDZ domain and CPAF_i_. (**D)** PDZ domain residue Arg^172^ makes cation-π and van der Waals interactions with CPAF_i_ residues Phe^260^ and Leu^264^ respectively. **(E)** PDZ residue Ser^176^ forms hydrogen-bonding interactions with CPAF_i_ residue His^267^.

Ligand recognition by the CPAF PDZ domain may play a critical role in activation of the zymogen during enzyme maturation. During chlamydial infection, CPAF is produced as a 67 kDa zymogen, which undergoes maturation by sequential autoproteolytic excision of a 40 amino acid inhibitory helix (CPAF_i_) that occludes the protease domain active site [28]. The inhibitory helix spans the 28 Å distance between the active site residue, Ser^491^, and CPAF^106-212^ (Fig 6A). In the cleft formed between the protease active site and the PDZ domain, the inhibitory sequence adopts a predominant alpha helical conformation and is followed by an N-terminal loop that is funneled along the PDZ binding site (Fig 6B and 6C). PDZ domain residue Arg^172^ is positioned to interact with Phe^260^ and Leu^264^ on CPAF_i_ through cation-π interactions and van der Waals interactions respectively (Fig 6D). Additionally, Ser^176^ forms hydrogen-bonding interactions with His^267^ on CPAF_i_ (Fig 6E). As a result, the C-terminal end of CPAF_i_ serves to block the proposed binding interface of the PDZ domain.

This orientation of the inhibitory sequence argues for a possible role in simultaneously preventing ligand binding to the PDZ domain in addition to blocking substrates from reaching the protease active site. Removal of the inhibitory sequence is required for enzyme activity and may be facilitated by the PDZ domain. We propose that, if the CPAF PDZ domain is involved in zymogen activation, one of several outcomes may occur upon cleavage at the first activation site between Met^234^ and Arg^235^: (1) substrate binding at the CPAF PDZ site induces conformational changes, similar to other S41 PDZ-containing proteases, after which the inhibitory sequence is presented to the active site in a catalytically competent conformation or (2) the singly cleaved inhibitory sequence obtains increased conformational flexibility, but remains bound to the CPAF PDZ domain to facilitate catalysis of the second and third cleavage events. Once the inhibitory segment is removed, the N- and C-terminal CPAF subunits associate to form the activated catalytic triad [28].

Interactions between the inhibitory helix and both the protease and PDZ domains highlight the potential for communication between the CPAF active site and PDZ domain. Such communication may form the basis for regulation of protease activation and/or substrate recognition. Distal regulation of protease activity is an established mechanism for the DegP and HtrA family hydrolases, as both classes exhibit PDZ-mediated oligomerization and activation [17, 20, 35, 40, 60]. It is possible that mutation of Arg^172^ and Ser^176^ may leave CPAF susceptible to hyperactivation as a result of activating PDZ ligands having easier access to the PDZ binding site.

Following activation of the CPAF zymogen and formation of the active enzyme, substrates are either cleaved in discrete positions or completely degraded, further demonstrating the broad activity of the enzyme. This catalytic duality suggests that CPAF may operate as both an endo- and exo-peptidase. These different modes of cleavage may be the result of active site pocket selectivity or may be linked to contributions from secondary substrate specificity binding sites. The dichotomy of CPAF proteolysis mechanisms and the lack of a rigid active site consensus motif suggest that CPAF targeting may be dictated by distal regulatory elements, one of which may be the putative, N-terminal PDZ domain.

The concept of PDZ domains as secondary substrate recognition sites in proteases is well established. The HtrA and DegP proteases utilize the PDZ domains to form higher order oligomers and as secondary binding sites for substrates [40]. In particular, DegP specifically has been shown to employ its PDZ domain in a “hold-and-bite mechanism” to guide the substrate into the protease domain active site [60]. Additionally, the *E*. *coli* tail-specific protease was shown to utilize a PDZ domain to assist in substrate binding. Mutations to the PDZ binding site resulted in a higher *K*_M_ and lower *k*_cat_/*K*_M_ for a peptide substrate [61]. Chlamydial HtrA (CT823) has also been shown to utilize one of the two PDZ domains for activation and oligomerization [62].

Because CPAF^106-212^ closely resembles human PDZ domains, we speculate that it may have evolved to hijack host protein-protein interactions, exposing them to potential CPAF-mediated proteolysis or directing CPAF to specific locales within the host cell. Alternatively, CPAF^106-212^ may intercept endogenous host PDZ ligands or PDZ-containing proteins to aid in molecular targeting of substrates. Others have shown that CPAF is released from the inclusion in late stages of infection [13], where it accrues in the cytosol and may displace endogenous host ligands. These findings would reveal new possible pathogenesis mechanisms for chlamydial effectors that use “zip-coding” strategies for targeting substrates and intracellular locales.

Many viral and bacterial pathogens have established a precedent of utilizing host-like PDZ domains or PDZ ligands as decoy pathogenesis effectors [21, 22, 26]. Viral proteins produced by vaccinia, avian influenza, human papilloma virus and herpes virus utilize PDZ-domains or PDZ ligand mimics and, upon binding, competitively abrogate the integrity of the natural host protein interactions [22, 26, 63]. Recent work has revealed disruption and degradation of epithelial tight junctions as an effective mechanism of viral pathogenesis [25]. More specifically, disruption of tight junctions affords a survival advantage for the virus in that enhanced penetration through epithelial barriers and trafficking to the basolateral membrane provides access to receptors capable of facilitating viral uptake. Likewise, *Chlamydia* infection disrupts tight junctions and adherens junctions in the single columnar layer of epithelial cells in genital tract and ocular infections, similarly to the sexually transmitted pathogen, *N*. *gonorrhoeae* [64].

CPAF^106-212^ has been shown to be similar to human Dlg, MPPx, MAG1, and SCRIB, PDZ-containing proteins, which are known to be components of cell polarity and tight junction assembly in epithelial cells. MPP7, for example, co-localizes in epithelial cells with tight junction proteins Dlg1, E-cadherin, occludin and CASK, and builds assemblies at the interface of the outer membrane that act as scaffolding for formation of tight junctions [65]. Chlamydial infections have been suggested to break down these junctions and disrupt epithelial cell integrity through degradation of nectin-1 [66] and keratin-8 and -18 [67], as well as sequestration of β-catenin and E-cadherin [68]. Such a mechanism would not be unique and has been described for *N*. *gonorrhoeae* [64]. Compromising the cellular junctions also allows for exposure of receptors on the basolateral membrane at these interfaces to other opportunistic pathogens such as HSV and HIV. Chlamydial disruption of host cellular junctions and the similarity of CPAF^106-212^ to known PDZ-containing junction proteins indicates that CPAF may be involved in disruption of epithelial cell junctions to promote chlamydial infection.

While CPAF may directly intercept host PDZ-mediated signaling, it may also indirectly influence additional PDZ-associated proteins. CPAF has been postulated to degrade nectin-1, a protein required for assembly of adherens junctions in epithelial cells [66]. We, and others, have demonstrated that CPAF is capable of degrading host cytoskeletal proteins vimentin and keratin 18 and nuclear envelope protein LAP1 [13–15, 69]. Interestingly, PDZ domain-mediated association of proteins with cytoskeletal machinery is plausible and has been observed for several proteins, including PDZ-GEF1, afadin, Dlg1, the PDZ-LIM family protein RIL and Lin-7 [70–74]. Because CPAF partially proteolyzes the vimentin cage surrounding the parasitophorous inclusion, we suggest that CPAF^106-212^ may facilitate localization of the enzyme to interact with vimentin, vimentin binding proteins, or associated cytoskeletal filaments. Vimentin has also been implicated as a component of epithelial tight junctions and may be a cellular target for CPAF proteolysis late in chlamydial infections [25]. Furthermore, vimentin and LAP1 interface with the inclusion vacuole by encapsulation of the vacuole and disruption of the nuclear envelope, respectively. CPAF utilizing a host-like PDZ domain to gain access to a host substrate directly or through scaffolding molecules would support previous data showing that the CPAF enzyme appears to be specific for at least two substrates, vimentin and LAP1.

It is interesting to note that several similar PDZ domains from *Mus musculus* and *Rattus norvegitus* were detected through our bioinformatic searches. The CPAF protein sequences between *C*. *trachomatis* and *C*. *muridarum* are highly conserved, suggesting that the *C*. *muridarum* may contain a homologous PDZ domain (S1 Fig). Furthermore, the proteins we detected in *M*. *musculus* and *R*. *norvegitus* were analogous to the Dlg proteins from *Homo sapiens*. These findings suggest that the *C*. *muridarum* CPAF PDZ domain may serve an analogous function in the infection and pathogenesis of *Chlamydia* in mouse and rat species. Because *C*. *muridarum* is a highly utilized, model pathogen for animal studies into chlamydial pathogenesis, investigations into the PDZ function using these tools would be highly beneficial.

## Conclusions

**Fig 7:**
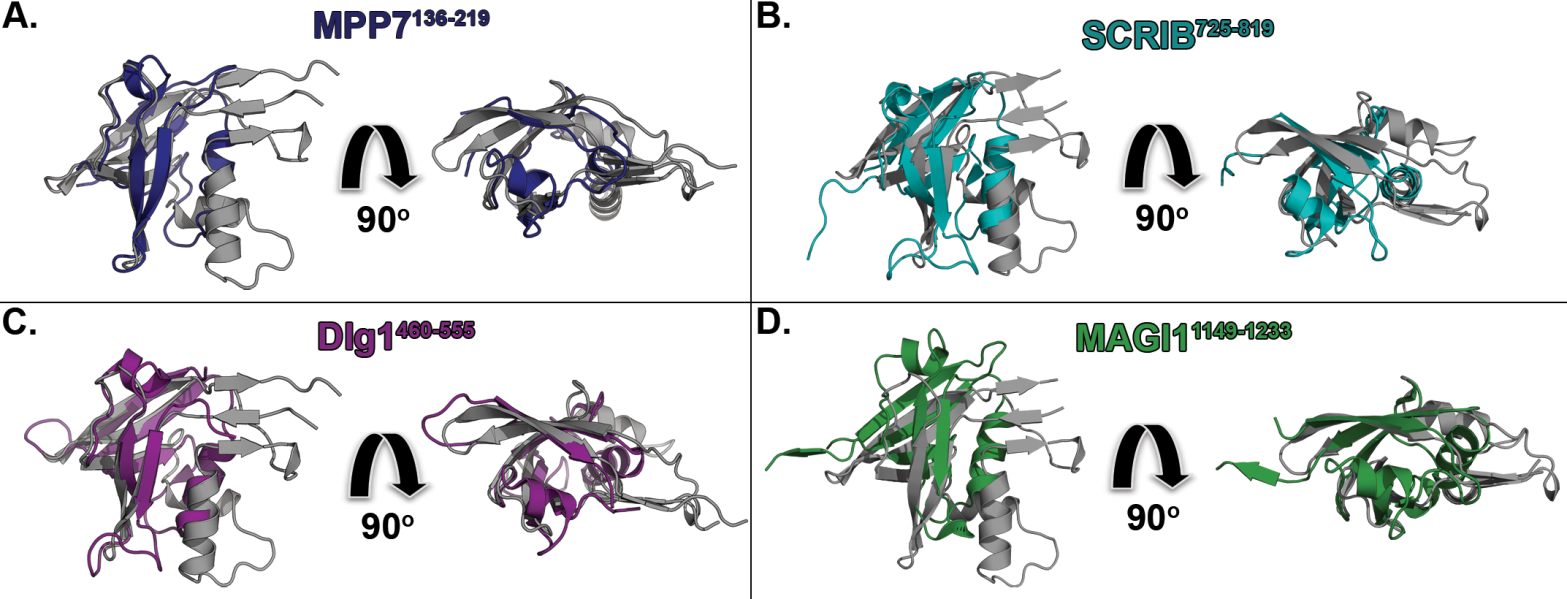
CPAF^106-212^ Bears Strong Structural Homology to Human PDZ Domains Involved in Epithelial Tight Junction Formation. The CPAF^106-212^ domain structure aligns well with the PDZ domain structures from human proteins **(A)** MPP7 (PDB ID: 3O46, blue), **(B)** SCRIB (PDB ID: 2W4F, teal), **(C)** Dlg1 (PDB ID: 1PDR, purple), and **(D)** MAGI1 (PDB ID: 2R4H, green) [75], which have been suggested to be involved in epithelial tight junction formation, cytoskeletal maintenance, and protein folding.

Bioinformatics analysis suggests that there exists a cryptic, host-like PDZ-domain found within the N-terminus of CPAF. Comparison of CPAF^106-212^ to canonical PDZ domain structures revealed that the CPAF PDZ bears structural homology to established PDZ classes and employs a unique YLPY-loop motif. Like the tail-specific HtrA and DegP proteases of the S41 family, we speculate that CPAF may utilize the PDZ domain as a molecular ruler to “hold-and-bite” target substrates. Further, the CPAF PDZ domain bears strong structural similarity to human-derived PDZ domains including Dlg1-4, MPP1, 5 and 7, and MAGI1, and SCRIB (Fig 7). Based on the similarity of the CPAF PDZ domain to host PDZ-forming sequences, we suggest that CPAF^106-212^ may be critical for proper cellular localization and to allow CPAF access to necessary substrates during infection. We propose that CPAF may utilize a PDZ domain for facilitating zymogen activation, cellular localization, secondary substrate recognition or a combination of these functions. Additional biochemical studies focusing on mutagenesis of critical PDZ residues, cellular localization, and domain swapping of CPAF^106-212^ will be key to elucidating the true role of the PDZ domain in the CPAF molecular mechanism of chlamydial pathogenesis.

## Supporting Information

S1 Fig Sequence Alignment of CPAF Homologues in Various *Chlamydia* Species. The CPAF106-212 region from *C*. *trachomatis* was aligned against the corresponding sequences from CPAF homologues across various chlamydial species. The abbreviations and UniProtKB identifiers for each CPAF variant are indicated. Highly conserved residues are shown in blue; absolute conservation is shown in red.
(TIF)

S2 Fig NCBI-BLAST Alignment Parameters from Comparison of CPAF^106-212^ to Host PDZ Domains. The E-values and bit scores for the results of the NCBI-BLAST alignment of CPAF^106-212^ against 170 candidate PDZ domains are shown with the E-value and bit score cutoffs shown in blue and red, respectively. Any results with E-value greater than 1 were deemed insignificant and removed from analysis. In the inset plot, the concentration of data points in the left, upper quadrant indicates a population of homologous sequences that exceed the cutoffs for both alignment parameters.
(TIF)
