# Supplementary figures and images for: The Chlamydia trachomatis Protease CPAF Contains a Cryptic PDZ-Like Domain with Similarity to Human Cell Polarity and Tight Junction PDZ-Containing Proteins
Source: PLoS One. 2016 Feb 1;11(2):e0147233. doi: 10.1371/journal.pone.0147233 (PMC4734761; doi:10.1371/journal.pone.0147233)

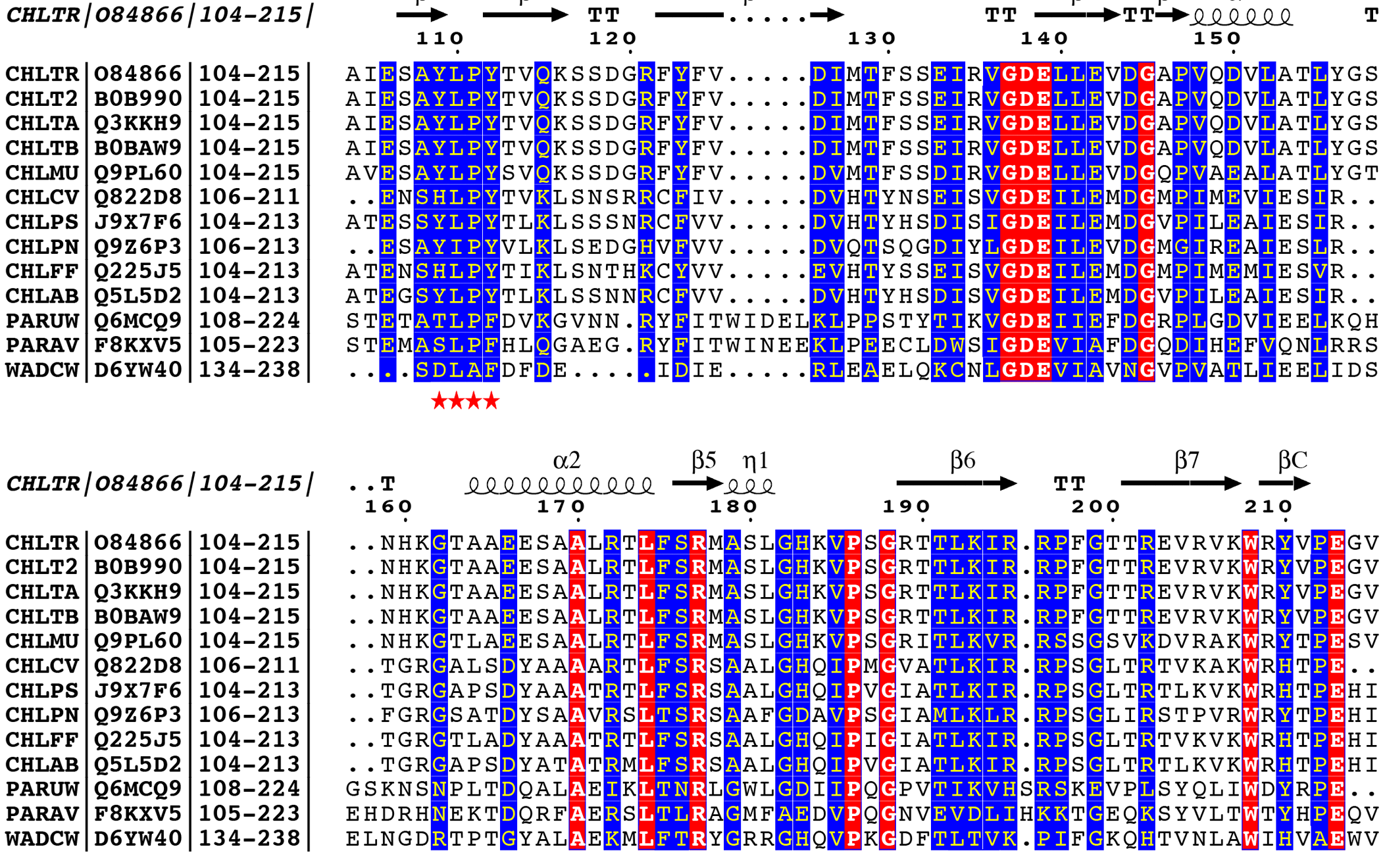

Supplement: S1 Fig — The CPAF106-212 region from C. trachomatis was aligned against the corresponding sequences from CPAF homologues across various chlamydial species. The abbreviations and UniProtKB identifiers for each CPAF variant are indicated. Highly conserved residues are shown in blue; absolute conservation is shown in red. (TIF) [file pone.0147233.s001.tif]

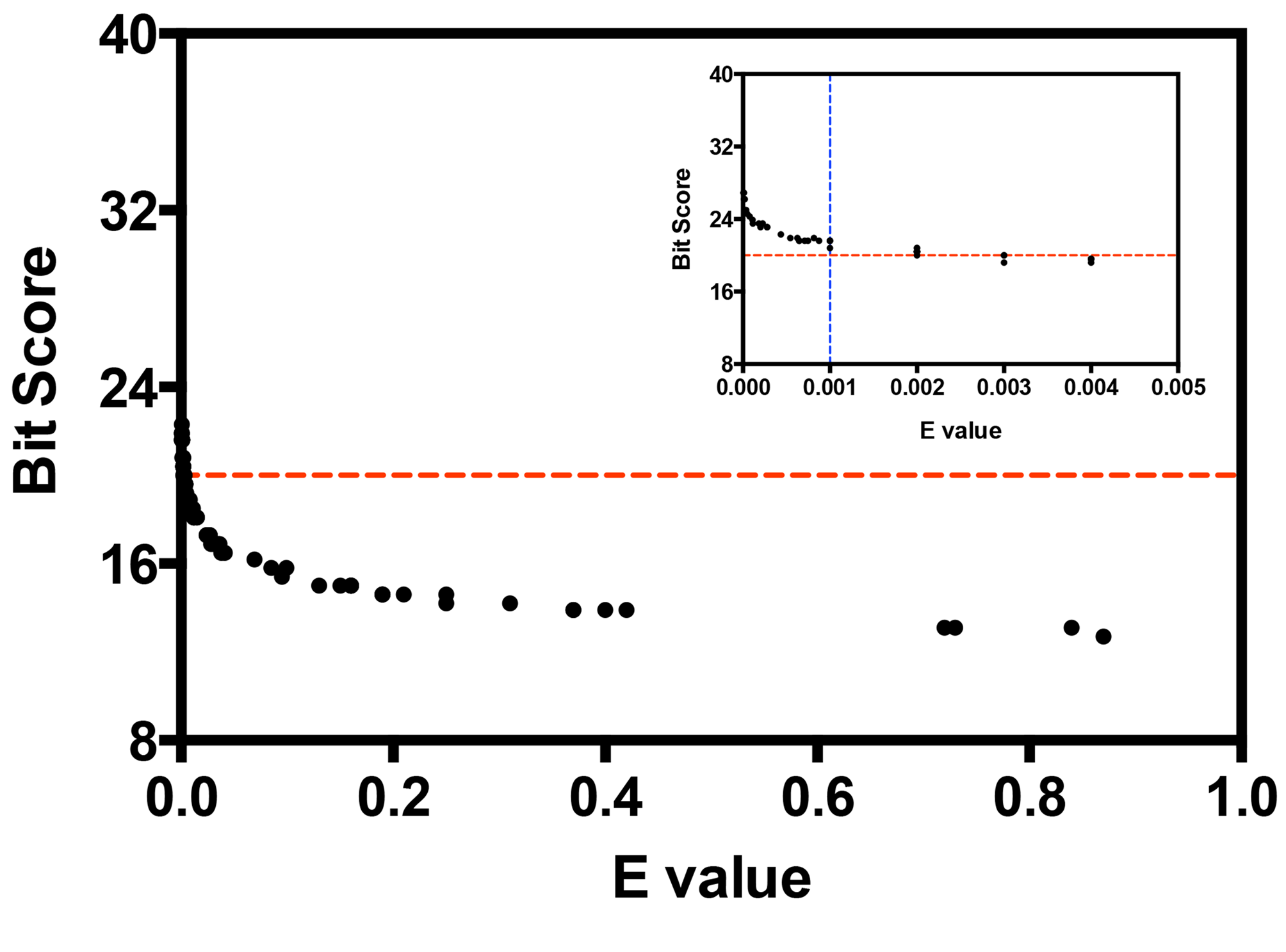

Supplement: S2 Fig — The E-values and bit scores for the results of the NCBI-BLAST alignment of CPAF106-212 against 170 candidate PDZ domains are shown with the E-value and bit score cutoffs shown in blue and red, respectively. Any results with E-value greater than 1 were deemed insignificant and removed from analysis. In the inset plot, the concentration of data points in the left, upper quadrant indicates a population of homologous sequences that exceed the cutoffs for both alignment parameters. (TIF) [file pone.0147233.s002.tif]
